# Evaluation of a blended-learning training concept to train oncology physicians to advise their patients about complementary and integrative medicine (KOKON-KTO): study protocol for a prospective, multi-center, cluster-randomized trial

**DOI:** 10.1186/s13063-019-3193-y

**Published:** 2019-01-29

**Authors:** Stefanie M. Helmer, Alizé A. Rogge, Felix Fischer, Daniel Pach, Markus Horneber, Stephanie Roll, Claudia M. Witt

**Affiliations:** 1 Institute for Social Medicine, Epidemiology, and Health Economics, Charité – Universitätsmedizin Berlin, corporate member of Freie Universität Berlin, Humboldt-Universität zu Berlin, and Berlin Institute of Health, Berlin, Germany; 2 Department of Psychosomatic Medicine, Center for Internal Medicine and Dermatology, Charité – Universitätsmedizin Berlin, corporate member of Freie Universität Berlin, Humboldt-Universität zu Berlin, and Berlin Institute of Health, Berlin, Germany; 3 Department of Internal Medicine, Division of Oncology and Hematology, Paracelsus Medical University, Klinikum Nuremberg, Nuremberg, Germany; 4 Institute for Complementary and Integrative Medicine, University of Zurich and UniversityHospital Zurich, Zurich, Switzerland; 5 Center for Integrative Medicine, University of Maryland School of Medicine, Baltimore, MD USA

**Keywords:** Cancer, Complementary medicine, Integrative medicine, Adverse effects, Communication, Blended learning

## Abstract

**Background:**

Many cancer patients are interested in complementary and integrative medicine during and after regular cancer treatment. Given the high number of users it is important that physicians and patients engage in a dialog about useful complementary and integrative medicine therapies during cancer treatment.

In a prospective, multi-center, cluster-randomized evaluation study we will develop, implement and evaluate a training program for oncology physicians advising their patients on complementary and integrative medicine. The main objective of the study is to evaluate whether training physicians in a blended-learning approach (e-learning + skills-training workshop) in providing advice to their cancer patients on complementary and integrative medicine, in addition to handing out an information leaflet about reputable websites, has different effects on the outcomes of patients, physicians, and their interaction level, compared to only giving out the information leaflet.

**Methods/design:**

Forty-eight oncology physicians will be included into a cluster-randomized trial to either participate or not in the blended-learning training. Physicians will then advise 10 cancer patients each, resulting in 480 patients participating in the trial. The blended learning consists of nine units of up to 45 min of e-learning and 18 units of up to 45 min of on-site skills-training workshop focusing. Outcomes will be measured on the physician, patient, and physician-patient-interaction level.

**Discussion:**

A blended-learning program for oncology physicians to advise their cancer patients in a systematic way and a reasonable time frame on complementary and integrative medicine will be evaluated in depth in a large cluster-randomized trial.

**Trial registration:**

German Clinical Trials Register, ID: DRKS00012704. Registered on 28 August 2017.

**Electronic supplementary material:**

The online version of this article (10.1186/s13063-019-3193-y) contains supplementary material, which is available to authorized users.

## Background

Complementary and integrative medicine (CIM) interventions have received increasing amounts of attention and are often used as self-management techniques for patients hoping to positively influence their cancer treatment [1, 2]. Motives mentioned by many patients were improving the immune system and increasing the effectivity of cancer treatment, reducing side effects and generally taking on an active part in their own treatment process [1, 3]. In line with this, some data show that the use of CIM during cancer care has increased consistently worldwide from only 25% of patients using it in the 1970s to 49% after 2000 [4].

Often patients face the variety of available CIM therapies without professional advice. The evidence for many CIM therapies is absent or inconclusive and new treatment forms appear frequently, highlighting the importance that patients get valid and understandable information. However, although patients wish to receive CIM information from their physicians [3, 5], many independently seek suitable treatments via numerous online sources. The uncovered full spectrum of faulty, unclear or evidence-based information may affect patients when making decisions in favor of, or against, CIM [6, 7]. To achieve the optimal course of treatment, patients hence should discuss their interest in CIM with their oncology physician so that patients’ expectations towards CIM can be taken into account during consultations and the use of CIM can be brought into line with cancer treatment [3, 8, 9].

Despite the necessity of it, many patients do not disclose their CIM use to their oncology physician [1, 10, 11]. Reasons for this can be explained on both sides of the patient-physician relationship. On the one hand, patients often do not find it necessary to discuss CIM with their respective physician, thus underestimating the harms or risks of interaction with their cancer treatment [12, 13]. Furthermore, patients anticipate and experience the physician’s disinterest in the topic and a negative response [14]. On the other hand, physicians might feel uncomfortable when providing information about evidence of CIM treatments [15]. Qualitative research in this field has shown that oncologists often regard the concept of CIM as being well outside their field [16]. However, health professionals in the field of oncology are often confronted with this topic. Therefore, the majority realize the great importance of being well informed in this area [15, 17].

Physicians’ willingness to be trained on CIM approaches shows that training programs in this area are strongly required [15]. A cluster-randomized pilot trial by our group showed that training physicians in providing advice during CIM-designated consultations scores highly for patient satisfaction and may be especially beneficial for physicians less experienced in CIM. However, physicians have reported that an additional consultation about CIM is time-consuming and that including long CIM consultations into everyday practice poses a problem [18].

To the best of our knowledge, a structured training program for oncology physicians in advising cancer patients via feasible consultations about CIM has not been established nor has there been a rigorous evaluation of CIM training at different levels (physicians, patients or their interaction) in Germany.

The Competence Network Complementary Medicine in Oncology (KOKON) is a collaborative research project in Germany (www.kompetenznetz-kokon.de). Funded by the German Cancer Aid, KOKON has aimed to improve knowledge and information transfer for CIM in oncology since 2012. This paper describes the KOKON-KTO study as part of the KOKON Competence Network.

The main objective of this study is to evaluate whether training physicians using a blended-learning approach (e-learning + workshop) to provide advice to their cancer patients on CIM, in addition to handing out an information leaflet about reputable websites, has different effects on the outcomes of patients, physicians and their interaction level, compared to only giving out the information leaflet.

## Methods/design

### Design

In a prospective, multi-center, cluster-randomized, two-armed study, 48 physicians will be included and randomized by cluster (hospital departments or private practices) into two groups, one that will be trained (intervention group) and the other that will not be trained (control group).

In the intervention group, oncology physicians will provide CIM advice to 10 cancer patients each and provide an information leaflet on reputable websites to their patients while oncology physicians in the control group will provide an information leaflet to their 10 recruited patients without giving additional advice on CIM.

As this study uses an exploratory approach, no sample size has been formally calculated. Based on logistical considerations, the anticipated physicians’ learning curve and in order to be able to give indications for further studies in this field, recruiting 400–480 patients seems reasonable.

### Physician recruitment and eligibility

The oncology physicians participating in the KOKON-KTO study will be recruited from hospital departments treating cancer patients (e.g., breast centers, departments for gynecology, ear, nose, throat departments, etc.) and private practices. Oncology physicians in private practices are part of the German health care insurance system and treat patients who are statutorily and privately insured. The described recruitment sites are hereinafter referred to as “hospital departments” and “private practices.”

From each hospital department two oncologists will have to be eligible and will be counted as a cluster. The 48 oncology physicians (24 working with specialization on gynecologic oncology) will be recruited from hospital departments (50%) and private practices (50%).

To allow heterogeneity, half of the participating oncology physicians will have a focus in gynecological oncology, while the other half will have focuses in other cancer entities. In Germany, gynecology is one of the surgical specialties of medicine. However, gynecology also includes a wide range of conservative treatment methods, such as hormone therapy, counseling, contraception methods, and reproductive health. All physicians with a specialization in gynecology participating in this study will have to work with cancer patients on a regular basis in hospital departments or private practices.

Recruitment will take place in the following federal states in Germany: Baden-Württemberg, Bavaria, Berlin, Hamburg, and North Rhine-Westphalia. Heads of hospital departments as well as representatives of private practices will be contacted by letters. The heads of hospital departments will be invited to send two oncology physicians each and the representatives of private practices will be invited directly to participate. Oncology physicians will be screened for their eligibility after receiving the study documents and subsequently will be included in the study on a “first-come first-served” basis.

Oncology physicians will be eligible if they fulfill the following selection criteria (based on self-reported information): specialization in the field of oncology, little knowledge of CIM, no previous structured trainings in CIM in the field of oncology, only a little experience in advising cancer patients on CIM, sufficient resources to conduct 10 consultations with cancer patients during their usual working hours, ability to take part in the on-site skills-training workshop in Berlin as well as good German language skills. Physicians will provide written informed consent to the study team. Physicians will retain the right to withdraw from the intervention and the study at any time during the course of the study. However, the study team will contact physicians to document the reasons for withdrawal.

Physician of both groups will receive 34 CME points by the German Doctors Association after the successful completion of the KOKON-KTO training.

We decided not to include radiation oncologists in the study because of their different medical specialization and to reduce the variation in oncology settings.

### Patient recruitment and eligibility

Cancer patients interested in advice on CIM will be recruited by the participating oncology physicians following a first-come first-served principle. For recruitment purposes, the physicians will receive study information materials provided by the central study office. Patients will be eligible if they meet the following criteria: age ≥ 18 years, diagnosed with cancer, being treated in the participating hospital department/practice, the cancer treatment has been already planned or is ongoing, good German language skills, interested in the topic evaluated by the question: “Are you interested in getting information about complementary medicine within cancer care from your oncology physician?” Patients with cognitive impairments (based on the subjective assessment of the physician that complex information cannot be transferred) will be excluded.

During the trial period, all patients will receive their usual cancer treatment and supportive care by their oncology care team. CIM consultations will be conducted as an addition to patients’ planned or ongoing cancer therapy. Patients will give oral and written informed consent to their participating oncology physicians. Patients will retain the right to withdraw from the study at any time during the study. However, the study team will contact them to record reasons for withdrawal.

### Random allocation procedures

The cluster-randomization into both groups will occur on a hospital department or private practice level. Whereas each private practice will provide only one oncology physician (cluster 1), hospital departments will each provide two oncology physicians (cluster 2). Randomization of clusters will be stratified by type of center (hospital department or practice) and specialization (gynecology, other oncology) using an allocation ratio of 1:1. After fulfilling the inclusion criteria and obtaining written informed consent from all participating physicians as well as, if necessary the heads of hospital departments, centers will be randomized by the study team. The randomization list will be generated by a statistician not otherwise involved in the study using SAS, version 9.4 (SAS Institute, Cary, NC, USA). The list will be transferred into a secured database (Microsoft Office Access 2010; Microsoft Corporation, Redmond, WA, USA) and be hidden behind the interface so that it will not be accessible to anyone involved in the random allocation or treatment. The order of randomization for both strata (gynecologist and oncologists) will follow the first-come first-served principle.

### Intervention procedures

The intervention of the study consists of two phases for the intervention group: the first phase with the focus on the physician level and the second phase as an interaction between physicians and their patients.

Phase I: physicians of the intervention group will receive a blended-learning training, which should qualify them to provide structured advice to their cancer patients on CIM in a KOKON-KTO consultation. The training and its content (knowledge about CIM and the KOKON-KTO consultation manual) have been developed in collaboration with KOKON project partners, taking findings of a previous pilot study [18] and published structured recommendations for discussing CIM in cancer care [19] into account. The subsequent development process included international experts and stakeholder engagement as well as peer review and testing phases. With the aim to increase feasibility and integration into daily medical work, the consultations were designed not to exceed a time frame of 20 min.

The KOKON-KTO training consists of the following components (Appendix):

1. KOKON-KTO e-learning: the KOKON-KTO e-learning contains information about CIM and the evidence and safety aspects of various treatments. Knowledge about therapies and supporting evidence, as well as safety aspects, will be measured by multiple-choice tests based on predetermined learning objectives. Moreover, the systematic KOKON-KTO consultation will be taught by using video sequences, audio and text information. The e-learning consists of nine obligatory lessons (up to 45 min each) and can be completed in approximately 6–7 h
2. Workshop: a 2-day on-site skills-training workshop consisting of 18 lessons (up to 45 min each) will train practical skills and implement the theoretical knowledge to provide CIM information in an empathic and systematic way to the patient. Exercises include dealing with unclear or missing evidence and typical, authentic challenges for preparing decision-making about CIM. Using a variety of teaching methods (e.g., role playing, consultations with standardized patients) typical consultation situations based on clinical case vignettes will be trained. Every workshop participant will practice a complete KOKON-KTO consultation about CIM in cancer care with a standardized patient

Learning objectives: after the KOKON-KTO training, oncology physicians should be able to compare different CIM and other supportive therapies and to lead a KOKON-KTO consultation adapted to context. After the KOKON-KTO e-learning oncology physicians should be able to (1) classify the needs and challenges of CIM in oncology, (2) differentiate between various CIM and other supportive therapies and (3) apply essential elements of the KOKON-KTO consultation in examples. After the on-site skills-training workshop, oncology physicians should be able to (1) apply knowledge on CIM and other supportive therapies, (2) implement elements of the KOKON-KTO conversation in role plays, (3) deal practically with the challenges of CIM and other supportive therapies, and (4) conduct a KOKON-KTO consultation about CIM with standardized patient.

The information leaflet that physicians will hand over to patients was developed by a multi-professional expert team (psychologists, oncologists, public health researcher) with knowledge of CIM in oncology, who selected three websites (https://www.kokoninfo.de/komplementaermedizin, https://www.krebsinformationsdienst.de/behandlung/unkonv-methoden-index.php, https://www.onkopedia.com/de/my-onkopedia/guidelines) providing information in the German language and one international website written in the English language (https://www.mskcc.org/cancer-care/diagnosis-treatment/symptom-management/integrative-medicine/herbs). All recommended websites provide information on CIM treatments, their possible applications and effectiveness, as well as their safety (e.g., interaction with cancer treatments). The information leaflet displays details on language, level of CIM education, and level of information provided. This way, patients will be able to choose the website most suitable for them.

Furthermore, a criteria list will be prepared that can be handed over by oncology physicians to patients to enable the latter to select trustworthy providers of CIM. This list will be developed in a modified expert consensus procedure involving multiple stakeholders. It will be made available to oncology physicians in the intervention group to use it within the KOKON-KTO consultations and can be handed out optionally to patients.

Phase II: after the training, each physician will conduct consultations following the KOKON-KTO consultation manual with 10 of their own patients. After each KOKON-KTO consultation, physicians will provide their patients with the information leaflet for reputable websites about CIM in oncology.

For the control group, the intervention of this study will consist of three phases:

- Phase I: physicians of the control group will receive instructions delivered via KOKON-KTO e-learning on how to provide the information leaflets for reputable websites about CIM in oncology to their cancer patients. The 45 min e-learning lesson will explain the handover of the leaflet and will describe possible challenges that can occur during this short consultation
- Phase II: physicians of the control group will provide 10 of their recruited patients with the information leaflet along with a short face-to-face consultation (2–5 min) about the content of the leaflet
- Phase III: after completion of the follow-up assessment of patients, the control group will receive the same blended-learning KOKON-KTO training. Afterwards, the control group will be asked to advise patients on CIM. Qualitative interviews will be subsequently conducted with all physicians after having KOKON-KTO consultations

**Fig. 1:**
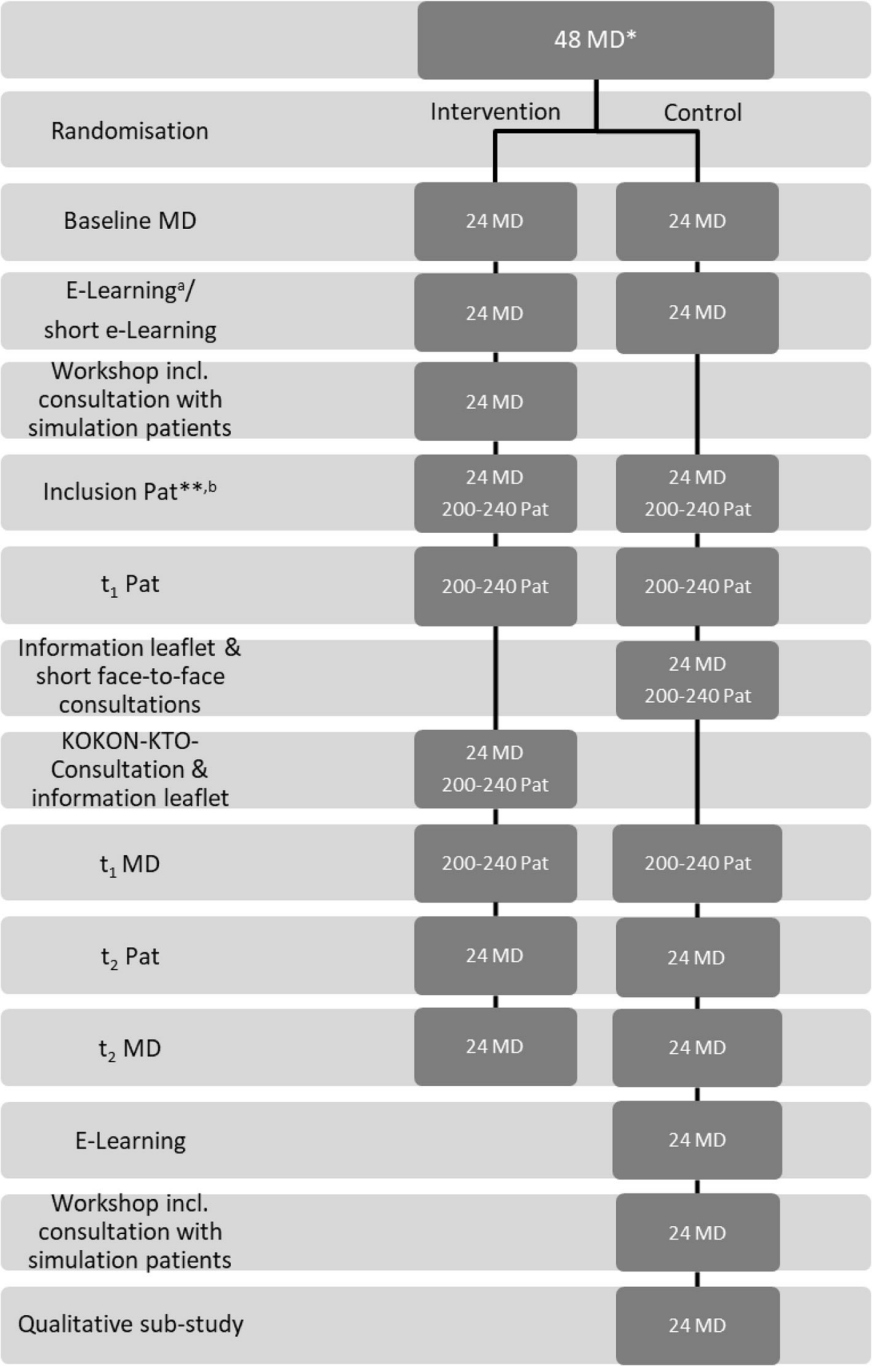
KOKON-KTO study flow chart. ^a^Includes questions about knowledge about complementary and integrative medicine (CIM). ^b^Includes provision of medical information by MD. *Oncology physicians (MD, medical doctor), **Patients

Both groups of physicians will be trained for the study procedures prior to the study. Furthermore, physicians will be trained at several time points (KOKON-KTO e-learning, skills-training workshop, KOKON-KTO consultation manual, study materials) for whom the KOKON-KTO consultation is appropriate. The oncology physicians will, therefore, learn that the KOKON-KTO consultation is only suitable for patients who show an interest in CIM and for whom the wish to use CIM is not an expression for another need such as fear of death or the need for information on cancer treatment. The study flow chart is shown in Fig. 1.

### Quantitative outcomes

Outcomes are based on an international consensus process including a workshop (Berlin, February 2017) and written rounds. During the workshop experts agreed that a single primary outcome would not be sufficient to capture all relevant information. Therefore, several outcomes on three different levels were suggested (physician, patient, and physician-patient-interaction level). Then, main and secondary outcomes were selected for each level (Table 1).

**Table 1:** Outcomes

	Domain	Method of measurement	Metric	Time frame
Physician level				
Main outcome	Perceived consultation skill competency	NRS	Fully disagree (0) to fully agree (10)	Weeks 7–30
	Perceived stress reaction	NRS	Fully disagree (0) to fully agree (10)	Weeks 7–30
Further outcomes	Level of CIM knowledge in cancer care	Multiple-choice questions	5 options, multiple response	Enrollment, Week 6
	Expectations regarding the effectiveness of CIM	5-point Likert scale	Fully agree to fully disagree	Enrollment, Week 6
	Expectations regarding the side effects of CIM	NRS	very safe (0) to not safe at all (10)	Enrollment, Week 6
	Personal attitude towards CIM	Situational Judgment Test	5 options, 1 response	Enrollment, Week 6
	Personal attitude towards CIM after knowing the results of the Situational Judgment Test that are based on expert ratings	Situational Judgment Test	5 options, 1 response	Week 6
	Application of the project-developed communication manual about CIM in oncology	Multiple-choice questions	5 options, 1 response	Weeks 1–6
	Handling challenges in consultations about CIM therapies	Situational Judgment Test	5 options, 1 response	Enrollment, Week 6
	Implementability of the manual-based consultation in the treating oncology physicians’ daily work	6-point Likert scale	Very good to not at all	Weeks 7–30,
	Duration of the CIM consultation	Time	Minutes	Weeks 7–30
	Reasons for communications longer than 20 min	Open-ended question		Weeks 7–30
Patient level				
Main outcomes	Communication between physician and patient.	EORTC-QLQ-COMU26	Not at all (1) to fully agree (4)	Week 2
	Patient satisfaction	PS-CaTE	Not at all (1) to fully agree (5)	Week 2
	Preparation for decision-making	PrepDM	Not at all (1) to very much (5)	Week 2
Further outcomes	Knowledge of CIM in cancer care	Multiple-choice questions	5 options, 1 response	− t1, Week 2
	Use of the recommended reputable websites about CIM in cancer care and subjectively experiences usefulness of the website	NRS	Very helpful (0) to not helpful at all (10)	Week 2
	Physician’s attitude towards CIM	NRS	Fully disagree (0) to fully agree (10)	Week 2
	Use of CIM and subjective therapeutic success	Multiple-choice questions	3 options: positive, negative, not sure	Week 2
Physician-patient-interaction level				
Main outcomes	Performance during communication with standardized patients	NRS	Fully disagree (0) to fully agree (10)	Week 7
	Interactive and communicative competencies	MAPI	Fully disagree (0) to fully agree (5)	Week 7

*CIM* complementary and integrative medicine, *EORTC-QLQ-COMU26 EORTC* communication module, *MAPI* Munich Physician Patient Interaction Inventory, *NRS* numerical rating scale, *PrepDM* Preparation for Decision Making, *PS-CaTE* Patient Satisfaction with Cancer Treatment Education

In addition, Situational Judgment Tests, a method of contextual competence diagnostics in a realistic context, will be developed and used as part of the KOKON-KTO e-learning for physicians in the control group. Excerpts from case vignettes will be used to describe specific challenges in the physician-patient interaction when discussing CIM. The given situation will be assessed by the participants selecting the most appropriate behavioral option(s) for them [19].

The main outcomes at the physician level are the perceived consultation skill competency (time frame: weeks 7–30) and the perceived stress reaction (time frame: weeks 7–30) in a consultation situation, which will be measured by two self-developed standardized instruments tailored to the specific KOKON-KTO consultation situation. The perceived consultation skill competency will be measured for three predefined emotional situations, which could occur during a consultation about CIM (overburden, tension, and discomfort with the consultation situation). Physicians will rate their perceived consultation skills on a numerical rating scale (NRS) from 0 to 10. Physician’s perceived stress reactions in a consultation situation will be measured on a NRS 0–10 as well.

Main outcomes (Table 1) on the patient level (time frame: week 2) will be as follows: issues related to the communication between the patient and their treating oncology physician will be measured by the EORTC communication module (EORTC-QLQ-COMU26) [20], which is intended to assess communication between patients and health professionals. It includes 26 items, which mainly assess behaviors related to communication and is organized into six scales and four individual items. To measure patient satisfaction with the information given on cancer treatment, two scales of the “Patient Satisfaction with Cancer Treatment Education (PS-CaTE)” questionnaire in German developed by the British Columbia Cancer Agency will be used [21]. Satisfaction with information regarding vitamins, herbs and complementary therapy as well as satisfaction with information sources and the that way information is provided will be measured on a 5-point Likert scale (“strongly disagree” to “strongly agree”). Furthermore, the “Preparation for Decision Making (PrepDM)” questionnaire [22] will be used to evaluate decision processes related to the preparation of patients for decision-making and engaging in dialogue with their practitioners. The 10-item questionnaire includes questions like: “Did this educational material help you recognize that a decision needs to be made?” and “Did this educational material prepare you to make a better decision?”

The main outcomes at the physician-patient-interaction level (Table 1) are the performance during communication with standardized patients (time frame: week 6). Oncology physicians participating in the study will undergo a KOKON-KTO consultation with standardized patients trained for study purposes. The KOKON-KTO consultation is intended to closely follow the KOKON-KTO consultation manual. Hence, the performance will be measured by a self-developed inventory tailored to this manual. Furthermore, interactive and communicative competencies will be rated using the “Munich Physician Patient Interaction Inventory (MAPI)” [23], which asks, for instance, whether the physician let the patient speak out or created a friendly consultation atmosphere. Both inventories will be rated by two independent raters who are present at the consultation with a standardized patient without influencing the interaction. Furthermore, qualitative interviews will be conducted as face-to-face-conversations with standardized patient to gain deeper insight into patient perspective.

### Demographic and clinical information and further variables

Physicians will be asked to supply details regarding their age, sex, level of occupational qualification, and number of years of experience with cancer patients. Furthermore, they will be asked to answer a questionnaire about the cancer treatment of each of the patients included in the study (current diagnosis, time since diagnosis, status of diagnosis, present and planned cancer treatment, intention of cancer treatment).

Patients will be asked about their past use of CIM and about the empathy of their physicians [24].

### Quantitative data collection

All physicians will be asked to answer questionnaires at several time points. First, by using a web-based survey prior to the e-learning, oncology physicians will provide their baseline information. Second, the physicians will complete a paper-pencil questionnaire after each communication with/handout of the information leaflet to 10 of their patients. Third, each physician will answer a paper-pencil questionnaire after completing the intervention with all 10 patients. Furthermore, physicians will be asked to evaluate the blended learning and to provide medical information about each patient after including them in the study. Patients will complete paper-pencil questionnaires at baseline (prior to the consultation/handout of the information leaflet) and 2 weeks after the consultation/handout of the information leaflet. For the physician-patient-interaction level, a systematic external rating by two independent raters will be conducted during the on-site skills-training workshop. For this, a consultation with a standardized patient will be conducted.

In the case of no or little recruitment, the study team will contact the physicians to offer information and support on how to improve recruitment or how to integrate the consultation into daily routine.

**Fig. 2:**
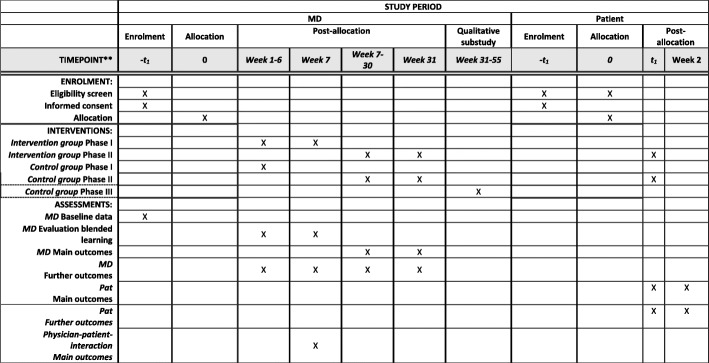
Content of the KOKON-KTO schedule of enrollment, interventions, and assessments according to the Standard Protocol Items: Recommendations for Interventional Trials (SPIRIT) Statement. *Oncology physicians (MD, medical doctor), **Patients

A Standard Protocol Items: Recommendations for Interventional Trials (SPIRIT) Figure and Checklist for this study protocol are provided in Fig. 2 and Additional file 1, respectively.

### Data management, storage and security

The data will be managed according to the Standard Operating Procedures (SOPs) and the quality management protocol of the coordinating center (Institute for Social Medicine, Epidemiology, and Health Economics, certified according to DIN EN ISO 9001:2015). An identification number (ID), not containing aspects that allow identification (e.g., initials or date of birth) will be assigned to each study participant (medical doctors (MDs) and patients). Data collection, data entry and data processing will be based on this ID. The data from the paper questionnaires will be entered in this pseudonymized form into a database programmed by a data manager of the Institute for Social Medicine, Epidemiology and Health Economics of the Charité – Universitätsmedizin Berlin. All data will be stored for at least 10 years. The archiving will be pseudonymized. The data will be collected using SoSci-Survey software and written questionnaires. In e-Learning (Moodlerooms), questions will also be asked in order to ascertain the development of the participants’ level of knowledge and experience. Within the scope of the study, only anonymized data will be evaluated. The data from the online survey will be transferred as CSV files and stored in a database at the coordinating center. The telephone interviews will be recorded with a digital sound-recording device, transcribed anonymously and stored using the ID.

### Statistics

All statistical analyses will be considered exploratory. Suitable summary statistics and graphical methods will be used to describe characteristics of the sample at baseline. Mean values and standard deviations (median and percentiles, if applicable) will be calculated for metric variables, frequencies and percentages for categorical variables.

Differences in the outcomes between the two treatment groups will be analyzed by multilevel models (analysis of covariance (ANCOVA) type or logistic-regression-type model). Models will include the physician as a random (clustering) effect, the stratification variables including type of center (hospital department or private practice) and specialization (gynecology or other oncology) as fixed effects, and the respective baseline value (if available) as a covariate. The multilevel structure of the data is due to patients treated within a physician, and physicians working within a cluster. However, due to the small number of physicians per cluster (one to two), the cluster (center) will not be taken into the model. For the treatment effect, adjusted mean values with 95% confidence intervals will be reported. In addition, a blinded analysis of baseline data will be performed to determine relevant baseline differences between the intervention and control groups. For sensitivity analysis, we will repeat the aforementioned models with adjustment for these variables. Furthermore, whether a different intensity of e-learning use (e.g., different increases in knowledge, duration of use) affects the outcomes (“dose-response relationship”) will be investigated. All *P* values will be considered explorative. A detailed statistical analysis plan (SAP) will be developed prior to any data analysis. Data analysis will be performed in SAS Version 9.4 or higher (SAS Inc., Cary, NC, USA) or SPSS Version 23 or higher.

### Qualitative data collection

A qualitative substudy will complement the quantitative findings. Qualitative semi-standardized telephone or online video interviews will be conducted with all oncology physicians of the control group who experienced both conditions (consultation with and without KOKON-KTO training). Interviews will occur 8 to 10 weeks after the on-site skills-training workshop of the control condition. The qualitative data will allow a fuller picture of the consultation experience.

Moreover, face-to-face-interviews will be conducted with all standardized patients after the on-site skills-training workshop. They will be interviewed in one-on-one, face-to-face, semi-structured interviews with one investigator to approach and explore patients’ perspective during KOKON-KTO consultations.

### Qualitative analysis

Control oncology physicians will be asked questions focusing on their personal experience when giving advice to their patients before and after the training. Standardized patients will be asked about their experience when getting advice from oncology physicians during the KOKON-KTO consultations. Transcripts will be imported into MAXQDA [25]. Data will be analyzed through content analysis according to Mayring and Flick [26, 27], deductive and inductive coding strategies will be combined. Deductive codes are predefined by the research team according to the semi-structured interview guideline. The coding of other subcategories of data analysis will follow a continuous process of extracting codes from conducted interviews and discussions among the team members. Patients’ quotes will be translated from German to English. Conducted interviews will be interpreted by two qualitative researchers in order to approximate reliability. Differences in the interpretation of the data will be discussed with the study team.

### Ethics

The study will be performed in agreement with the Declaration of Helsinki and has been approved by the relevant local ethics boards (Ethics Committee of Charité – Universitätsmedizin Berlin, Medical Association Hamburg, Medical Association Baden-Wuerttemberg, Medical Association Nord Rhine, Ethics Committee of the Medical Association of Westphalia-Lippe, Ethics Committee at the Medical Faculty of Würzburg, Ethics Committee of the Medical Faculty of Heidelberg, Ethics Commission of the Albert-Ludwigs-University of Freiburg). In addition, issues regarding data protection were approved by the local data protection officer at Charité – Universitätsmedizin Berlin. All study participants will provide oral and written informed consent.

Physicians will have to spend a considerable amount of time for the study including e-learning, workshop, assessment, and KOKON-KTO consultations. There is a risk that oncology physicians will feel overwhelmed by the training and study procedures. A user-centric approach will be used for the study documents and the KOKON-KTO blended learning. An experienced team will prepare the respective materials.

Standardized consultations in workshops or role plays can be perceived as stressful by some physicians and physicians in the control group may be burdened by the fact that they will only hand over a flyer with recommendation for reputable websites within a short consultation without a communication training. However, physicians in the control group will be prepared for this situation in a 45-min e-learning.

Patients will have to invest time for the study to complete questionnaires and there might be patients who will be tempted to use alternative strategies which interfere with their cancer treatment. To prevent these, patient assessments will be minimized. Moreover, physicians will be trained to inform about evidence-based CIM approaches and that CIM treatment, if used, should be complementary and not alternative to cancer treatments. Furthermore, they will mention, where applicable, the risks of interactions.

### Governance, quality assurance, and safety

This study is part of a larger network project (Competence Network Complementary Medicine in Oncology) that is managed by a steering group that consists of seven members. The principle investigator of this project is also a member of this steering group. Relevant design changes and safety issues as well as progress of the project according to predefined milestones will be discussed in the steering group that has monthly conference calls. Furthermore, the network project has an advisory board that can also provide advice for this trial.

The trial will be conducted according to the Standard Operating Procedures (SOPs) and the quality management protocol of the coordinating center (Institute for Social Medicine, Epidemiology, and Health Economics, certified according to DIN EN ISO 9001:2015). Data quality will be checked by a data manager following SOPs. The institute receives annual audits according to DIN EN ISO 9001:2015.

### Dissemination

We aim to publish the study results in international peer-reviewed medical journals. Moreover, we plan to present results nationally and internationally at oncology, gynecology, and CIM conferences and to provide website information.

## Discussion

This trial will help to determine the effects of a blended-learning training for a CIM consultation with cancer patients. We will evaluate outcomes at the physician-patient-interaction level. The study and the training that we aim to evaluate contain several unique aspects. First, this is a large cluster-randomized trial that will investigate a structured training on this topic for oncology physicians in Germany. Furthermore, the consultation about CIM in cancer care should not be longer that 20 min so it can be integrated into usual care. One of our previous studies demonstrated that oncology physicians found it difficult to integrate stand-alone time-intensive CIM consultations into their daily routine. However, physicians also stated that they found the additional time to talk with their patients satisfying [19]. Therefore, our concept will address this challenge. To overcome it, we will tailor the training to the needs of oncology physicians when giving advice to their own patients. This will make the consultation less time-consuming.

Another unique point is the broad investigation of effects on different levels. We will ask not only oncology physicians about their opinion on the training and the consultations but also the patients. Furthermore, we will analyze the physician-patient interaction as this was a major recommendation produced by our expert workshop on outcomes.

Our study protocol has several distinctive aspects including that cancer patients not only obtain their cancer care in hospital departments but also in private practices in Germany. We aim to include both types of institutions and will ask oncology physicians with varying specializations (oncology as well as gynecology) to participate in our study. Moreover, physicians from five different federal states will be asked to participate. In addition, we will include only CIM-inexperienced physicians in our study. Based on our previous pilot study we assume that they might gain a greater benefit from training on communicating with patients about CIM [18].

This study is subject to limitations. First, it should be mentioned that a 20-min consultation is not sufficient to cover all possible issues arising around CIM in oncology as well as other challenges that might occur during this consultation. The focus of this study is to train physicians for a more general approach that can be easily implemented in their daily medical work. For CIM consultations that are more complex and might have a different focus, existing guidelines and other settings can be applied [28].

Even though the consultations and the consultation manual were planned carefully and after consideration of expert opinions, it cannot be guaranteed that the discussion will be applicable to cancer patients in practice and under real-life conditions without restrictions. However, these doubts should be dispelled by the testing of the KOKON-KTO consultation manual by experienced oncology physicians who are unfamiliar with the training in advance. Furthermore, oncology physicians participating in the KOKON-KTO study will have exemplary consultations with standardized patients before conducting consultation with their own patients. The KOKON-KTO training will be the first to be tested in a randomized controlled trial with patients under real-life conditions.

There is a risk that oncology physicians in the control group may feel overwhelmed in the consultation situation due to the low level of CIM training. However, in routine care they frequently receive questions on CIM by their cancer patients. Being able to provide a leaflet might not solve the situation but perhaps improve it. The interviews and the completion of the interview assessments will take place during the working hours of the physicians. In the previous study, no aspects that we would have categorized as side effects of the consultations were documented. Future studies might think about appropriate safety measures for this type of interventions.

In addition, the study will not examine whether the physicians actually adhere to the manual and training and follow the recommendations when conducting their consultations. However, e-learning sections on indication-specific CIM therapies are intended to increase the knowledge about effectiveness and safety and, therefore, the quality of CIM advice in the KOKON-KTO consultations. Furthermore, the on-site skills-training workshop including a standardized patient setting will train the oncology physicians to adhere to the KOKON-KTO consultation manual. Neither physicians nor patients can be blinded regarding group allocation because of the different training content and consultation settings, and bias due to this cannot be excluded.

As another limitation of the study, the KOKON-KTO training, consisting of a 6-h e-learning and a 2-day on-site skills-training workshop, is relatively time-consuming and is, therefore, only suitable for physicians who are interested in the topic and in giving CIM advice to patients.

With regard to the measurement of the data, mainly self-administered questionnaires with inherent limitations will be applied. Moreover, the consultations conducted by the oncology physicians with standardized patients will not be recorded on video but merely assessed by a systematic external rating. This procedure results from our previous study [18], in which the participating oncology physicians were partially disapproving of video recordings. KOKON-KTO consultations will only be observed and objectively rated during the workshop at the end of the training. This study will not include radiation oncologists because of their different oncology setting. This way it is easier to develop the materials targeted to the treatment situation for our blended learning. For future trainings these materials might be added and evaluated.

In the case of questions regarding patient’s satisfaction with the consultation, ceiling effects may also occur, which have already occurred in our previous study [18]. Therefore, it might be important to get a better understanding of the meaning of satisfaction for this type of patients. However, since we will use outcomes on several levels we decided not to include a broader evaluation of satisfaction.

Our study will provide evidence on a training program (e-learning + workshop) for oncology physicians on advising their patients on CIM, and the study will offer data on the program’s effects in comparison to only handing out an information leaflet about reputable websites. Furthermore, the study will not only provide new information about the effects on outcomes at the physician level but also at the patient, and the patient-physician-interaction level.

## Trial status

The trial is currently in the process of recruiting. Recruitment of physicians started in June 2017 and was completed in December 2017. Patient recruitment started in April 2018.

### Additional file

Additional file 1: Standard Protocol Items: Recommendations for Interventional Trials (SPIRIT) 2013 Checklist. (DOC 124 kb)

## Appendix

**Table 2:** Curriculum of the KOKON-KTO training

Source	Course title	Content	Time (units)
e-learning	Complementary medicine for cancer	• Definition of CIM • Use, needs, and challenges of CIM in oncology	1
	CIM therapies	• Whole medical systems • Nutrition • Exercises • Mindfulness • Phytotherapy • Vitamins and supplements	6
	KOKON-KTO consultation	• KOKON-KTO consultation manual • Example videos	2
Workshop	Day 1	• Role plays • Impulse lectures • Exchange of experience • Audio statements • Discussion rounds • Mistletoe	8
	Day 2	• Challenges arising during CIM consultations by observing two role plays with an expert as consultation-leading physician • Nutrition • Standardized KOKON-KTO consultation	8

*CIM* complementary and integrative medicine
